# Damping Properties of Large-Scale Overlap Joints Bonded with Epoxy Hybrid Resin or Polyurethanes: Experimental Characterisation and Analytical Description

**DOI:** 10.3390/polym15051102

**Published:** 2023-02-22

**Authors:** Jannis Damm, Matthias Albiez

**Affiliations:** KIT Steel- and Lightweight Structures, Karlsruhe Institute of Technology (KIT), 76131 Karlsruhe, Germany

**Keywords:** adhesively bonded overlap joint, damping properties, adhesive damping, loss factor, experimental investigations, dimensional analysis, steel structures

## Abstract

Structures in various industries are exposed to dynamic loads. The dissipative properties of adhesively bonded joints can contribute to the damping of dynamically stressed structures. Dynamic hysteresis tests are carried out to determine the damping properties of adhesively bonded overlap joints by varying the geometry and test boundary conditions. The dimensions of the overlap joints are full-scale and thus relevant for steel construction. Based on the results of experimental investigations, a methodology is developed for the analytic determination of the damping properties of adhesively bonded overlap joints for various specimen geometries and stress boundary conditions. For this purpose, dimensional analysis is carried out using the Buckingham Pi Theorem. In summary, it can be stated that the loss factor of adhesively bonded overlap joints investigated within this study is in the range between 0.16 and 0.41. The damping properties can particularly be increased by increasing the adhesive layer thickness and reducing the overlap length. The functional relationships of all the test results shown can be determined by dimensional analysis. An analytical determination of the loss factor considering all identified influencing factors is enabled by derived regression functions with a high coefficient of determination.

## 1. Introduction

### 1.1. Damping Properties of Adhesives and Adhesively Bonded Joints

A large number of structures in different branches are exposed to dynamic loads. These include the construction sector [1] as well as automotive and mechanical engineering [2,3]. Damping measures are often required to reduce the resulting oscillations. Adhesively bonded joints can be an alternative to conventional measures such as vibration dampers and absorbers.

Adhesives based on epoxy–resins or polyurethanes can be modified in such a way that they have good damping properties in addition to very good strength properties. The viscoelastic material behaviour of the adhesive creates material damping, which can contribute to the damping within a structure.

In order to determine the damping properties of adhesive layers and adhesively bonded joints, several analytical and numerical approaches have been developed and published in the past decades. Important publications on the damping properties of adhesively bonded overlap joints are summarised in the following.

The mechanical properties of polymers can first be determined on bulk samples. Representative work in this area, especially for epoxy–resins, has been published by Chen et al. [4] and Wei et al. [5]. The study of the types of heat release under mechanical stress of elastomers modified with multi-walled carbon nanotubes was published by Shchegolkov et al. [6]. Among other things, investigations were carried out using a Raman Microscope and DSC. It is shown that the release of energy depends, among other things, on the temperature and the tensile strain.

The mechanical and damping properties of polymers can change as soon as they are used in composite specimens. An accurate description is complex. In early works, He and Rao [7,8] developed analytical models for calculating the free vibration behaviour of two-dimensional, one- and two-section jointed beam elements articulated on both sides using the energy method and Hamilton’s principle. The adhesives are implemented in the calculations by using complex modules as a viscoelastic material. Based on this, He and Rao [9] carry out numerical calculations in further works on the basis of the analytical calculation approach developed. The influence of the joint geometry and variable material parameters on the natural modes, the associated natural frequencies and the loss factor are investigated.

Kaya et al. [10] extend the existing calculation models by a third dimension. In addition, the influence of different geometry and material parameters on the eigenmodes as well as the resonance amplitudes of three-dimensional, unilaterally clamped, single-overlap joints are investigated by means of numerical calculations.

Almitani and Othman [11] develop an analytical model to analyse the influence of viscoelastic adhesive layers as well as varying geometry parameters on the dynamic response behaviour of axial, predominantly shear-loaded, single- and double-overlap joints. The substrates consist of both metallic and polymer materials. The investigations focus on the influence of the loss factor of the adhesive on the maximum vibration amplitudes as well as on the average and maximum shear stresses within the investigated test specimens in the range of the natural frequencies.

Investigations into the damping behaviour of adhesively bonded, multilayer composite structures for use in aircraft, spacecraft, transport vehicles and mechanical systems are the subject of the work by Yaman and Şansveren [12]. The focus of the investigations is on the optimisation of adhesive bonding with regard to the stiffness and energy dissipation capacity of structures joined with different types of adhesive joints. In addition, the influence of various geometric parameters such as adhesive layer thickness, overlap length, number of layers and fibre orientation on the natural frequency and the damping properties is investigated both numerically and experimentally.

In summary, the presented work is primarily limited to the development of analytical models for the description of the damping properties of plane overlap joints on a scale that is less relevant for steel construction. The focus of this paper is, therefore, on the experimental investigation of the damping properties of adhesively bonded overlap joints on the scale of steel structures. In addition, the influence of relevant geometric and test parameters on the damping properties of the test specimens is investigated and discussed within the context of a parameter study.

The use of adhesives and adhesively bonded joints as an alternative to conventional joining techniques in steel construction is within the focus of current research. For example, tubular joints and structures [13,14,15,16] and offshore applications [17,18,19] are relevant to the current research. Furthermore, the results of investigations on the damping properties of adhesively bonded circular hollow section joints are presented in another paper by the authors [20].

The potential for damping dynamically stressed steel structures by adhesively bonded joints has already been shown in works by Damm et al. [21,22], Vervaeke et al. [2] and Rao [3]. For the dimensioning and design of dynamically loaded steel structures, the reliable estimation of the existing damping under consideration of the adhesively bonded joints is of high relevance.

### 1.2. Scope of This Paper

The scope of this paper is set on the experimental investigation of the damping properties of semi-structural adhesives based on epoxy-hybrid resin and polyurethane and adhesively bonded joints manufactured with them. Numerical studies by Damm et al. [4] show that the dynamic behaviour of steel structures with adhesively bonded joints can be significantly optimised by considering adhesive-inherent damping. Due to the high relevance for applications in many sectors, the focus of this paper is set on the damping properties of adhesively bonded single-overlap joints. The focus in the experimental investigations of large-scale bonded overlap joints is set on a predominant shear stress state within the adhesive layer. The method, results and analysis presented in this paper were published for the first time in reference [22] in German.

The dimensions of the overlap joints are chosen based on real steel applications and are thus relevant for steel construction. Furthermore, geometry and testing conditions are varied as part of a parameter study. The objective is the knowledge of the influence of different geometry parameters (adhesive layer thickness, overlap length) as well as test boundary conditions (stress level, frequency) on the damping properties of adhesively bonded overlap joints.

So far, there are no models for the prediction of this local joint damping that can be used in the context of a practical design of steel structures. For this reason, the aim of this paper is to summarise the experimental results of dynamic tests of adhesively bonded overlap joints and to analyse and analytically describe the functional relationship of the experimental results presented by means of dimensional analysis.

## 2. Materials and Methods

### 2.1. Materials and Samples

#### 2.1.1. Adhesives and Their Experimental Characterisation

Within the study, the epoxy–resin hybrid-based 2-component adhesive Sikadur F51-60 (referred to as adhesive EPH) from SIKA AG^®^ and the semi-structural 2-component polyurethane adhesive Körapur 842 (referred to as adhesive PUR) from Kömmerling Chemische Fabrik GmbH^®^ are characterised with regard to their mechanical properties under static and dynamic loading on bulk and bonded specimen. The experimental investigations for the characterisation of the adhesives are carried out within the framework of the research project P 1272 [23] at the Laboratorium für Werkstoff- und Fügetechnik (LWF) of the University of Paderborn. Both adhesives are suitable for application by injection due to their low viscosity. The applicability of the adhesives on corundum-blasted surfaces can be verified by tensile tests on tensile shear and head tensile specimens documented in [23]. Supplementary information can be found in Göddecke et al. [24].

#### 2.1.2. Overlap Joints Made of Steel Substrates

The specimen type of the single-overlap joint (referred to below as overlap joint) consists of two steel plates with the dimensions L_S_ and B_S_ and the sheet thickness t_S_, which are adhesively bonded with a defined overlap length L and a defined adhesive layer thickness t_adh_. The substrates of the overlap joint consist of flat steel products of grade S355J2, according to DIN EN 10058 [25]. The base material of the steel profiles has a yield strength of 355 MPa and a tensile strength of 490 MPa, according to DIN EN 1993-1-1 [26]. Young’s modulus is 210,000 MPa.

**Figure 1:**
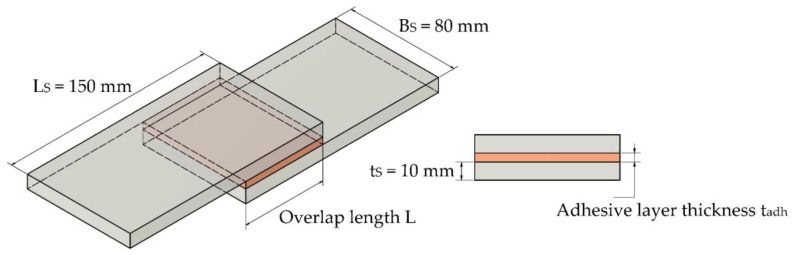
Schematic representation of the overlap joint showing relevant geometric dimensions of the substrates and the adhesive layer in accordance with [22].

A schematic representation of the overlap joint and details of the relevant geometric dimensions are documented in Figure 1. The values given for the adhesive layer thickness are nominal values.

Prior to bonding, the surfaces to be bonded are cleaned of coarse dirt and cleaned with methyl ethyl ketone (MEK). Subsequently, bonded contact surfaces are blasted over a large area with corundum screening to the surface quality Sa 3 according to EN ISO 8501-1 [27]. The blasted surfaces are then cleaned again with MEK.

### 2.2. Experimental Investigations of Adhesively Bonded Overlap Joints

#### 2.2.1. Experimental Series

In the first series of tests, the static load–deformation behaviour of the bonded overlap joint is determined using quasi-static tensile tests. The determined force–displacement curves are then converted into shear strength–shear strain curves [23].

Based on these results, shear–strain-based test levels are defined for the dynamic tests. The damping properties of the bonded overlap joints are determined in dynamic tests with external harmonic excitation. In this context, the effects of the following parameters on the damping properties of the tubular joints are investigated:

- Test frequency (1 Hz, 3 Hz, 5 Hz);
- Overlap length L (20 mm, 60 mm);
- Adhesive layer thickness t_adh_ (2.5 mm, 5.8 mm, 8.0 mm);
- Shear–strain level γ in the adhesive layer (2%, 8%).

#### 2.2.2. Manufacturing of the Adhesively Bonded Overlap Joints

**Figure 2:**
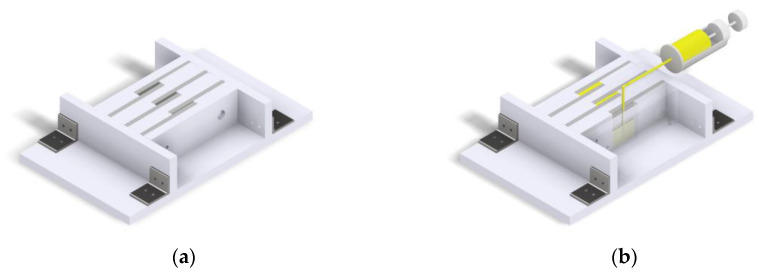
Adhesive bonding of the overlap joint in accordance with [22]. (**a**) Placing the blasted and degreased substrates into the production device. (**b**) Adhesive application by injection on the bottom of the joint gap until the adhesive emerges at the top of the joint gap.

For the manufacturing of the overlap joint, a manufacturing device made of polyethylene (PE) is used. The process steps of the adhesive manufacturing of the overlap joint are visualised in Figure 2.

First, all components of the manufacturing device are cleaned and degreased. Then, the manufacturing device is assembled. The manufacturing device is designed in such a way that different adhesive layer thicknesses and overlap lengths can be realised. Subsequently, the cleaned, blasted and degreased substrates are inserted into the manufacturing device (Figure 2a). The adhesive bonding of the substrates is achieved by injecting the adhesive into the joining gap. Depending on the thickness of the adhesive layer, the adhesive is injected from the upper side into the joining gap using a cannula or cartridge syringe (Figure 2b). The adhesive is applied from the bottom of the joining gap upwards. In this way, any air inclusions are transported to the top of the joint gap and do not remain in the adhesive layer. The tip of the cannula or cartridge syringe is continuously guided out of the joint gap while the adhesive rises in the joint gap. The injection process is completed as soon as the adhesive emerges from the top of the joint gap.

#### 2.2.3. Quasi-Static Tensile Tests to Determine the Load–Deformation Behaviour of Adhesively Bonded Overlap Joints

In order to determine the damping properties of the overlap joints under defined stress states, uniaxial, quasi-static tensile tests are first carried out to determine the load–deformation behaviour.

Experimental testing of the bonded overlap joint is carried out on a universal testing machine inspekt 250 kN in upright two-column design. The specimen is clamped on both sides by the hydraulic clamping jaws of the testing machine. At the upper clamping device, the clamping jaws move on both sides, whereby the specimen is clamped centrically. The test speed is 1 mm/min. The compensation of the specimen-related eccentricity takes place at the lower clamping device. During the execution of the static tensile tests, machine force and machine displacement are recorded.

The test specimen configurations 1 (t_adh_ = 2.5 mm) with the lowes<t adhesive layer thickness and 3 (t_adh_ = 8.0 mm) with the highest adhesive layer thickness are examined. In addition, the two overlap lengths, 20 mm and 60 mm, are examined for each test specimen configuration. As shown in [22], conclusions on the load–deformation behaviour for configuration 2 can be derived on the basis of the generated data.

The force–displacement curves are then converted into shear stress–shear strain curves using the specimen dimensions. The determined shear stress–shear strain diagrams are used as a basis for determining shear strain-based test levels. These allow the dynamic tests to be carried out under defined and uniform test boundary conditions.

#### 2.2.4. Dynamic Tests with Harmonic External Excitation to Determine the Damping Properties of Adhesively Bonded Overlap Joints

**Figure 3:**
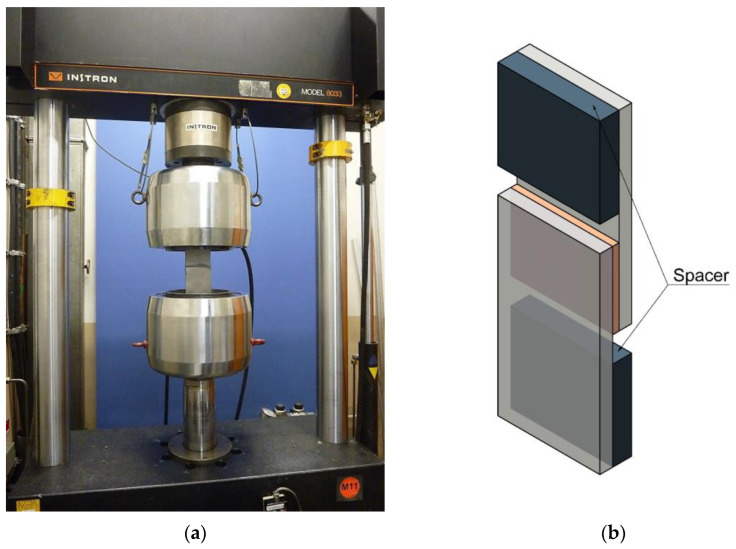
(**a**) Experimental set-up of the dynamic tests on adhesively bonded overlap joints [22]; (**b**) Schematic representation of an overlap joint as well as the spacers for connecting the specimen to the testing machine (coloured blue) [22].

The experimental testing of the adhesively bonded overlap joints is carried out on a servo-hydraulic 250 kN testing machine. The test set-up for the dynamic tests on bonded overlap joints is shown in Figure 3a. The dynamic tests are performed as hysteretic tests under alternating tension–compression.

As for the static tensile tests, the test specimen is clamped on both sides by the hydraulic clamping jaws of the testing machine. In addition, spacers of different thicknesses are inserted on both sides depending on the tested adhesive layer thickness. These serve to compensate for the eccentricity caused by the specimen, thus preventing the occurrence of clamping moments. The resulting axis of the test load runs centrally through the adhesive layer, which means that the adhesive layer is under predominant shear stress. Figure 3b shows the adhesively bonded overlap joint schematically. In addition, the spacers (blue) and their positioning during the test are visualised.

Test parameters and test series of the experimental investigations are summarised in Table 1. The focus of this publication is set on the results of experimental investigations of adhesive EPH. In addition, an exemplary comparison of the EPH and PUR adhesives is provided.

The dynamic tests are carried out on the basis of defined test levels A and B, which are characterised in Section 3.1. The test frequencies 1 Hz, 3 Hz and 5 Hz are applied in direct succession within both test levels. So, it is possible to determine the influence of the test frequency and the associated change in strain rate on the damping properties.

Machine force and machine displacement are documented over time. Up to 20 test cycles are recorded per test configuration. The sampling rate of the data acquisition software is set as a function of the test frequency and ranges from 1200 Hz to 4800 Hz to record the data [22].

#### 2.2.5. Analysis Routine

The damping properties of the adhesively bonded overlap joints are quantified using the loss factor η, which can be determined using Equation (1) [28].

(1) $$\eta =\frac{W_{d}}{2{\pi W}_{e}}=\frac{\psi }{2\pi }=\frac{\Delta }{\pi }=\tan\delta$$

with

η—loss factor
W_d/_W_e_—quotient of dissipated and elastically stored energy
Ψ—specific damping capacity
Δ—logarithmic decrement
tan δ—tangent of the phase angle δ

The loss factor can be determined by using both an energy-based and a time-based evaluation [29]. In this study, first, the time-based evaluation routine is used. Here, the loss factor is determined based on the time offset between excitation and response of a specimen due to harmonic excitation, which can be observed for viscoelastic polymers [28]. From this time offset, the phase angle δ of the two harmonic signals can be determined by Equation (2) and converted into the loss factor according to Equation (1). The factor f in Equation (2) corresponds to the test frequency.

(2) δ [°]=Δt·f·360°

Two tests are carried out for each test specimen configuration. Several load cycles are recorded for each test, from which an average loss factor is determined. The loss factor of the two tests carried out is then arithmetically averaged. The coefficient of variation (CV) of the results of the individual hysteresis is given as the mean value of the two tests carried out in the results. The evaluation of the test results is performed by using OriginLab 2020b. Before the evaluation, the test data are flattened to eliminate any existing measurement noise in the data [20].

The loss factor can be transformed into other known parameters for describing damping properties, such as the specific damping capacity ψ or the logarithmic decrement Δ [28]. The loss factor and the tangent of the phase angle δ are equivalent and of particular interest here. The tangent of the phase angle is determined in the context of a DMTA on adhesive bulk samples. This enables the comparison between the adhesive characterisation results documented in [20] and the results of the experimental investigations documented in this paper. Furthermore, the loss factor is used as a parameter specifically for describing the damping property of adhesives in a large number of international publications. These include, for example, the works of Vaziri and Nayeb-Hashemi [30,31] and Almitani and Othman [11]. The experimentally obtained data presented can thus be integrated into the existing data basis [22].

The experimental data presented in this paper is evaluated by means of the time-based approach explained above. Comparative observations with the energy-based evaluation method were carried out. The two routines provide almost identical results.

### 2.3. Dimensional Analysis of the Functional Relationship of the Results of the Experimental Investigation of the Damping Properties of Adhesively Bonded Overlap Joints

The objective of the dimensional analysis is to develop a methodology to identify the functional relationship of the results of experimental investigations on adhesively bonded overlap joints using the associated parameters, which have an influence on the loss factor of the overlap joints. In the following, they are therefore referred to as influencing factors.

It is often useful to represent the relationship between the associated influencing factors as simplified as possible. A corresponding representation can be developed by means of dimensional analysis. Here, a variety of examples of physical processes are formulated without dimensions. All relevant influencing factors are related to characteristic variables of the problem at hand, resulting in a dimensionless formulation.

The principle of dimensional analysis is based on the Buckingham Pi Theorem, published by Egard Buckingham in 1914 [32]. The requirement for the application of the Pi Theorem is the knowledge of the model for the description of a physical problem as well as the associated variables that influence this problem. If these requirements are given, the Pi Theorem can be applied [33]. According to [32], the solution of a physical problem can be represented by n independent influencing factors a_1_, a_2_, …, a_n_ with a total of k basic dimensions via Equation (3) [33].

(3) $$f\;\left(a_{1},a_{2},\ldots ,a_{n}\right)=0$$

Equation (3) can be expressed by n–k dimensionless and independent parameters П_1_, П_2_,…, П_n−k_ according to Equation (4) [33].

(4) $$F\;\left({П}_{1},{П}_{2},\ldots ,{П}_{n-k}\right)=0$$

It is assumed that there is no functional relationship between the influencing factors and that Equation (3) can be described as dimensionally homogeneous.

The dimensionless parameters Пi can be formed by a combination of the identified influencing factors, which is the central task of dimensional analysis. A reasonable combination of the influencing factors must be identified by means of experimental investigations. The Pi Theorem provides information about the number of dimensionless parameters and not about how they are composed or what relationship exists between them [33].

The required number of tests depends on the identified influencing factors and can be very large depending on the complexity of the relationship to be determined. For this reason, a functional correlation of test results can alternatively be approximated by iteratively combining the influencing factors and parameters [33]. This practical approach is followed in this paper and is particularly practicable for simpler correlations for which knowledge about the influence of the identified influencing factors already exists.

For the identification of the functional relationship, the parameters can be combined and exponentiated as desired. It is advisable to build up the combination of the parameters on the basis of known relationships. If a linear or quadratic influence of an influencing factor is already known in relation to the target variable, this can be taken into consideration directly in the dimensional analysis. In addition, care should be taken to simplify the functional relationship as much as possible [22,33].

## 3. Results

### 3.1. Load–Deformation Behaviour of Adhesively Bonded Overlap Joints under Quasi-Static Load

**Figure 4:**
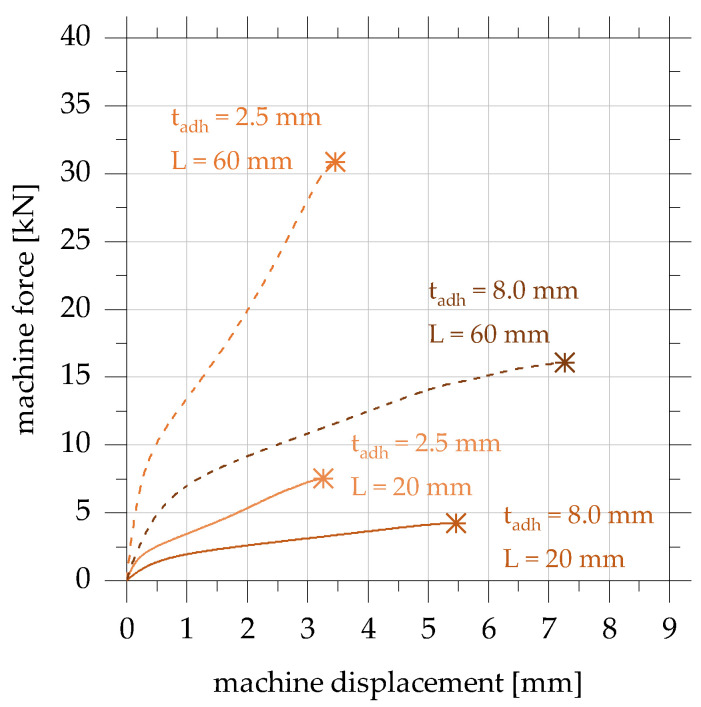
Machine force–machine displacement diagram of quasi-static tensile tests on overlap joints in accordance with [22].

The machine force–machine displacement curves (in short, force–displacement curves) are shown for the adhesive EPH in Figure 4. Due to the reproducibility of the test results, only one exemplary curve per test specimen configuration is shown. The point of maximum force is marked with an asterisk followed by an immediate load drop.

The force–displacement curves of the overlap joints manufactured with the adhesive EPH initially show across all geometries a linear increase up to a machine displacement of approx. 0.25 mm. Afterward, the force–displacement curves of all tested specimens flatten increasingly. From a displacement of between 0.5 mm and 1.0 mm, the force–displacement curves again show linear behaviour until the specimen breaks. However, the slope in this second linear range is clearly below the linear initial stiffness. At the same adhesive layer thickness, the curves of the specimens with an overlap length of 60 mm are, as expected, above those of the specimens with an overlap length of 20 mm. The load–bearing capacities of all the tests carried out range between 4.3 kN and 31.9 kN, depending on the geometry of the specimen.

**Figure 5:**
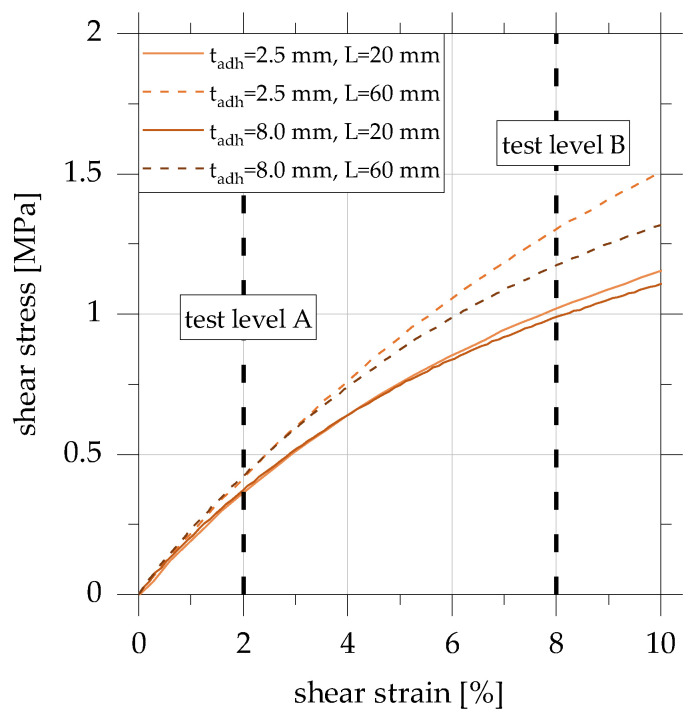
Shear stress–shear strain diagram of quasi-static tensile tests on overlap joints in accordance with [22].

The shear stress–shear strain curves derived from the force–displacement curves are shown in Figure 5 for a relevant range of shear strain up to 10%.

The shear stress–shear strain curves of the overlap joints bonded with the adhesive EPH show that the initial stiffnesses are approximately identical up to a shear strain of 1.5% for all specimen geometries considered. For the adhesive EPH, a significant deviation of the curves depending on the specimen geometry can be observed from a value of the shear strain of approx. 2% to 2.5%. From a shear strain of 3%, the curves flatten and then continue with a lower slope.

Two test levels, A and B, are derived on the basis of the previously shown shear stress–strain curves, which can be characterised as follows:

- Test level A: γ ± 2%, range of approx. linear initial stiffness and linear viscoelastic behaviour, low influence of non-linear material behavior,
- Test level B: γ ± 8%, end of the range of linear initial stiffness, decreasing joint stiffness depending on the adhesive layer geometry, the influence of non-linear material behavior.

### 3.2. Damping Properties of Overlap Joints Bonded with an Epoxy–Resin-Hybrid-Based 2-Component Adhesive

**Figure 6:**
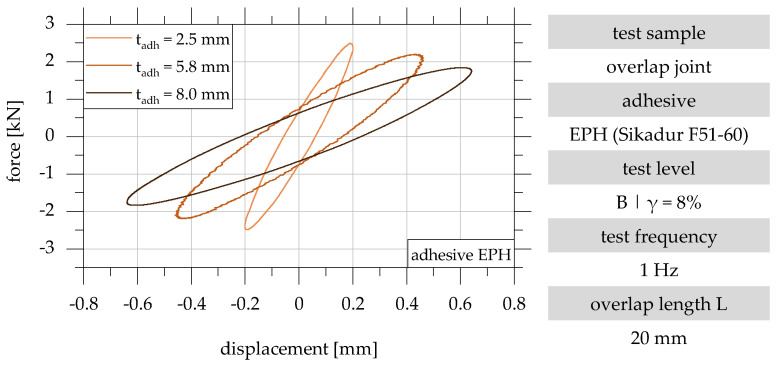
Force–displacement–hysteresis of the overlap joint configurations 1, 2 and 3 with an overlap length of 20 mm in test level B (γ = 8%) in accordance with [22].

The damping properties of adhesively bonded overlap joints are determined from the recorded force–displacement–hysteresis. Exemplary force–displacement–hysteresis of the overlap joint configurations 1, 2 and 3 with an overlap length of 20 mm in test level B (γ = 8%) are shown in Figure 6.

**Figure 7:**
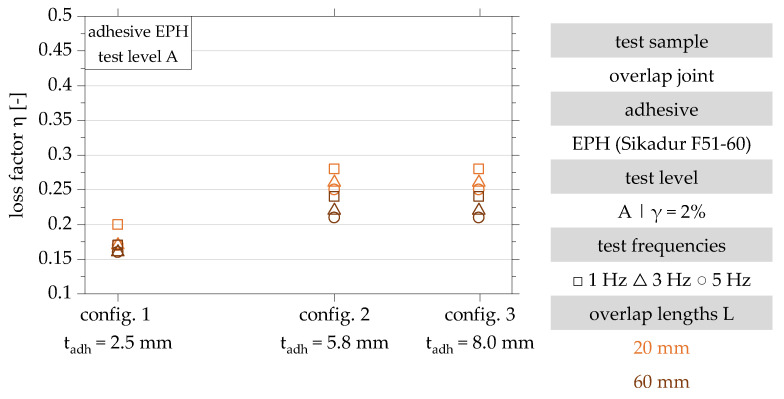
Results of the dynamic investigation of the damping characteristics of the overlap joint configurations 1, 2 and 3 for the determination of the loss factor in test level A (γ = 2%) in accordance with [22].

The results of the dynamic investigation of the overlap joint configurations 1, 2 and 3 in test level A are shown graphically in Figure 7 in the form of loss factors. The loss factors determined according to Section 2.2.5 are between 0.16 and 0.28. The lowest loss factor is achieved for configuration 1 and is independent of the overlap length. The maximum loss factor in test level A is 0.28 and results for configurations 2 and 3 with an overlap length of 20 mm.

**Figure 8:**
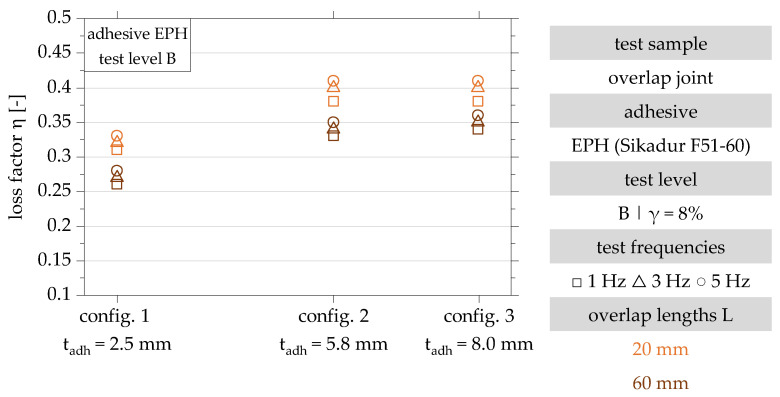
Results of the dynamic investigation of the damping characteristics of the overlap joint configurations 1, 2 and 3 for the determination of the loss factor in test level B (γ = 8%) in accordance with [22].

The results in test level A show a decrease in the loss factor with increasing test frequency for all configurations and both overlap lengths. An increase in the adhesive layer thickness, on the other hand, is associated with an increase in the loss factor for both overlap lengths. An asymptotic increase of the dissipation factor with increasing adhesive layer thickness is observed for both tested overlap lengths. Examination of the results for configurations 1 and 2 shows a clear increase in the loss factor with an increase in adhesive layer thickness. Increasing the adhesive layer thickness to 8.0 mm does not lead to a further significant increase in the loss factor for either of the overlap lengths investigated. The results of test level B are shown in Figure 8.

In test level B, with a minimum loss factor of 0.26 and a maximum loss factor of 0.41, an increase in the minimum and maximum loss factors can be observed with an increase in the maximum shear strain of the adhesive layer. In test level B, a change in the influence of the test frequency can be observed. A test of all specimen configurations with a maximum slip of 8% leads to an increase in the loss factor with increasing test frequency across all geometries [22].

Analogous to the results in test level A, an asymptotic increase of the loss factor with increasing adhesive layer thickness is also shown in test level B.

A summary of the loss factors determined for the overlap joint configurations 1, 2 and 3 depending on the overlap length, the test level and the frequency is given in Table 2. In addition, the coefficient of variation (CV) of the results of the individual hysteresis is given as the mean value of the two tests carried out. The calculated CV shown is between 0.02 and 0.13 and mostly below 0.05.

A comparison of the absolute loss factors in test levels A and B shows that the damping increases with an increasing shear strain of the adhesive layer and is, therefore, non-linear. The decrease of the loss factor with increasing overlap length can also be observed in test level B. In summary, the test results show that the damping properties of adhesively bonded joints can mainly be enhanced by increasing the adhesive layer thickness and reducing the overlap length. The test frequency is of minor importance.

## 4. Discussion

### 4.1. Influence of the Test Frequency

**Figure 9:**
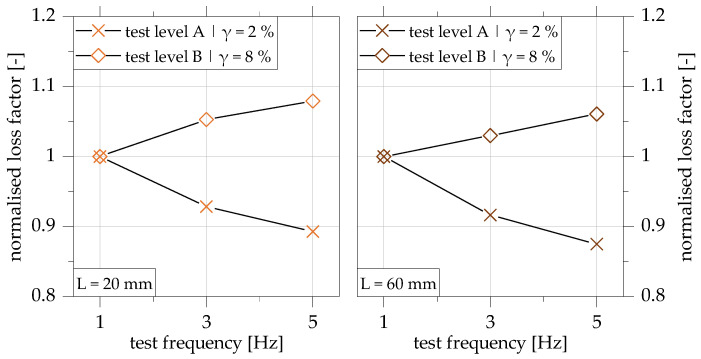
Influence of test frequency on the loss factor for overlap joint configuration 2 (t_adh_ = 5.8 mm) with overlap lengths of 20 mm (**left**) and 60 mm (**right**) in test levels A and B; plot normalised to the loss factor at test frequency of 1 Hz based on [22].

First, the influence of different test and geometric parameters on the damping properties is analysed and discussed. Figure 9 shows the analysis of the influence of the test frequency on the loss factor, exemplarily for configuration 2 with an adhesive layer thickness of 5.8 mm separately for the overlap lengths 20 mm and 60 mm for the two test levels A and B. The results are, therefore, converted into a normalised representation of the test frequency of 1 Hz.

In Figure 9, it can be determined that the frequency-dependent damping properties of the overlap joint are additionally influenced by the level of shear strain in the adhesive layer. In test level A, a decrease of the loss factor with an increase of the test frequency from 1 Hz to 5 Hz by more than 10% can be observed. This is applicable for both overlap lengths investigated. In test level B, on the other hand, there is an increase in the loss factor with increasing test frequency. In the frequency range considered, the loss factor normalised to 1 Hz increases almost linearly by more than 5% for both overlap lengths. The average change of the loss factor due to an increase of the test frequency from 1 Hz to 5 Hz is approx. 0.03 for both test levels.

The reason for the change of the frequency influence can be minor damage at the polymer level of the adhesives. The damage that takes place leads to a change in the polymer structure, which increases the internal friction on the polymer chain level. The damage that takes place leads to a change in the polymer structure, which increases the internal friction at the polymer chain level. This results in an increase in dissipated energy, which can explain the higher loss factor with increasing test frequency in test level B.

This hypothesis is supported by the observations in [34]. The authors associate fatigue-induced damage to the adhesive layer with an increase in the loss factor of adhesively bonded joints. In particular, the dissipative polymer properties are influenced by the progressive damage. The stiffness of the bonded joint is only negligibly affected, especially for low numbers of load cycles. Related studies are documented in [35]. Here, the authors correlate the damage to scarf joints with the increase in potential energy.

The shear stress–shear strain–hysteresis documented in [22,24] shows that any damage that may occur has no measurable effect on the stiffness and the maximum force reached in the recorded hysteresis.

Finally, it can be stated that the frequency level in the considered spectrum (1 Hz up to 5 Hz) is of minor importance from a practical point of view. The results of numerical investigations in [21] show that a change in the loss factor of significantly less than 0.05 is of negligible magnitude from a practical construction point of view. The investigation and discussion of the influence of the overlap length, the adhesive layer thickness and the adhesive are, therefore, carried out exemplarily for the relevant test frequency of 1 Hz.

### 4.2. Influence of the Overlap Length

**Figure 10:**
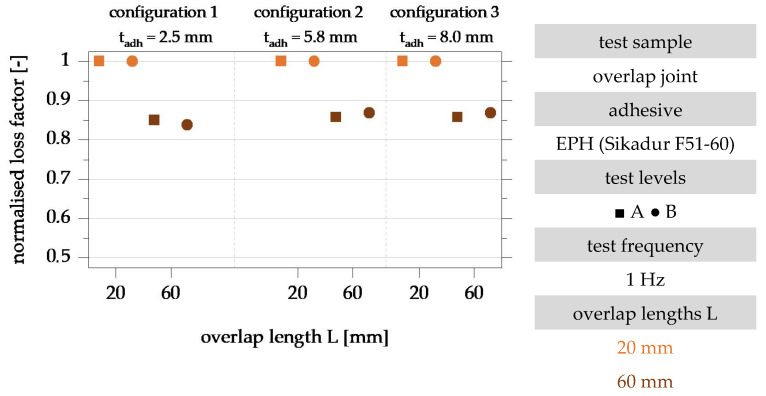
Evaluation of the influence of the overlap length on the loss factor of the overlap joint configurations 1, 2 and 3 with overlap lengths 20 mm and 60 mm in the test levels A and B for the test frequency 1 Hz; representation normalised to the loss factor of the specimens with overlap length 20 mm based on [22].

To evaluate the influence of the overlap length on the loss factor, the results for specimen configurations A, B and C with an overlap length of 60 mm are normalised to the results of the same configuration with an overlap length of 20 mm. The normalised evaluation of the test results is shown in Figure 10.

The evaluation of the normalised loss factors in Figure 10 shows a decrease in the loss factor with an increase in the overlap length for all investigated specimen configurations. The normalised loss factor of the specimens with an overlap length of 60 mm is between 84% (configuration 1, t_adh_ = 2.5 mm) and 87% (configuration 3, t_adh_ = 8.0 mm) and is 86% on average. The influence of the overlap length on the loss factor is, therefore, almost independent of the adhesive layer thickness. An analysis of the results in the two test levels also shows no correlation between the level of shear strain or stress and the detected overlap length influence.

The decreasing damping properties with increasing overlap length can be explained by the deformation state of the adhesive layer, which is dependent on the overlap length. The deformation of the substrates increases with increasing overlap length. The proportion of the joint part deformations in the applied deformation increases with increasing overlap length. This can explain the decrease in the dissipative properties of the bonded joint [22].

### 4.3. Influence of the Adhesive Layer Thickness

**Figure 11:**
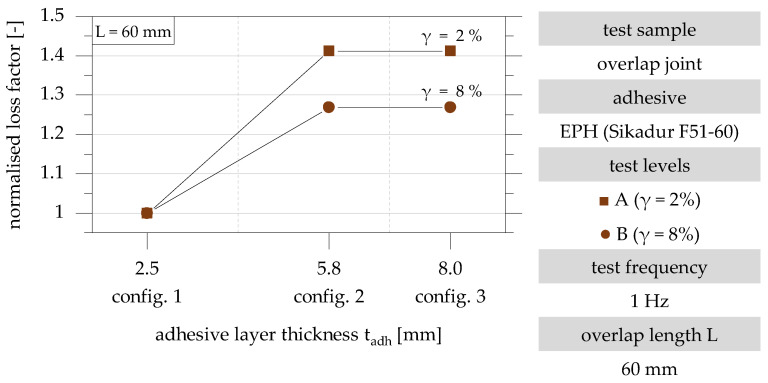
Evaluation of the influence of the overlap length on the loss factor of the overlap joint configurations 1, 2 and 3 with overlap lengths 20 mm and 60 mm in the test levels A and B for the test frequency 1 Hz; representation normalised to the loss factor of the specimens with overlap length 20 mm based on [22].

The basis for the discussion of the quantitative influence of the adhesive layer thickness on the loss factor of the overlap joint is the evaluation of the test results shown in Figure 11, normalised to the adhesive layer thickness of 2.5 mm. The evaluation is carried out separately for the two test levels, A and B.

Figure 11 shows an increase in the normalised loss factor for the adhesive layer thickness of 5.8 mm. The increase of the loss factor depends on the maximum shear strain of the adhesive layer and is 41% in test level A and 27% in test level B. The influence of the adhesive layer thickness, therefore, decreases with increasing shear strain of the adhesive layer. As a result of a further increase in the adhesive layer thickness to 8.0 mm, no further increase in the loss factor can be observed. This observation applies to both test levels investigated.

The increase in the loss factor with increasing adhesive layer thickness can be explained by adhesion forces occurring in the area of the joint component. In the area near the surface of the joint part, deformation of the adjacent adhesive layer is hindered. The substrates near the adhesive layer behave almost rigidly in comparison to the adhesive layer, whereby transverse contractions, as well as shear deformations in the area near to the part to be joined, are hindered. This means that the shear deformations in the area of the joint component are significantly lower than those in the center of the adhesive layer. A three-dimensional stress state develops near the boundary surface. The proportion of the adhesive layer whose deformation is not limited by adhesion forces increases with increasing adhesive layer thickness. The influence of adhesive forces decreases with rising adhesive layer thickness. The loss factor does not increase further as a result of a change in the adhesive layer thickness from 5.8 to 8.0 mm [22].

### 4.4. Influence of the Adhesive Used

**Figure 12:**
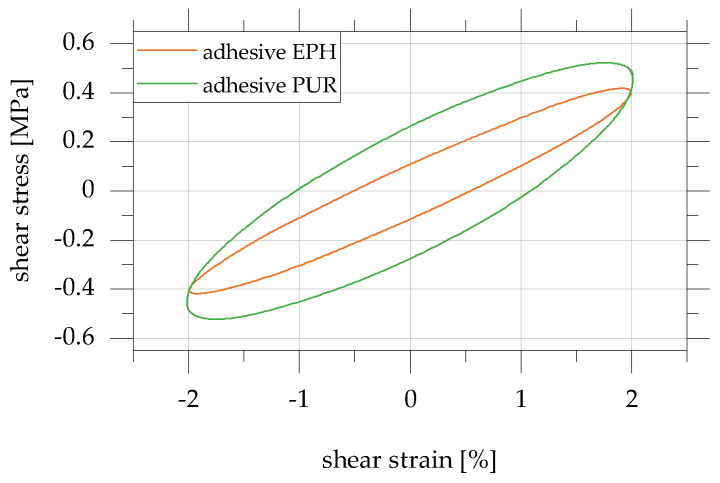
Analysis of the influence of the adhesive on the shear stress–shear strain hysteresis in test level A; exemplary representation for specimen configuration 2 with overlap length 20 mm for the test frequency 1 Hz for the adhesives EPH and PUR based on [22].

The results in test level A for the test frequency 1 Hz are used to discuss the influence of the adhesive on the damping properties of adhesively bonded overlap joints. Figure 12 shows the shear stress–shear strain hysteresis of specimen configuration 2 with an overlap length of 20 mm, which is used to analyse the principal differences in the damping and force–deformation behaviour of the overlap joints made with the EPH and PUR adhesives.

It can be seen that, in particular, the area of the hysteresis shown, and thus, the dissipated energy per load cycle is greater for the PUR adhesive (E_PUR_ = 356 MPa) compared to the EPH adhesive (E_EPH_ = 86 MPa). The area of the hysteresis assigned to the PUR adhesive with the higher modulus of elasticity is larger by a factor of 2.4. In contrast, the shear stress when reaching the maximum shear strain is significantly less influenced by the adhesive used (EPH: 0.4 MPa; PUR: 0.45 MPa).

**Figure 13:**
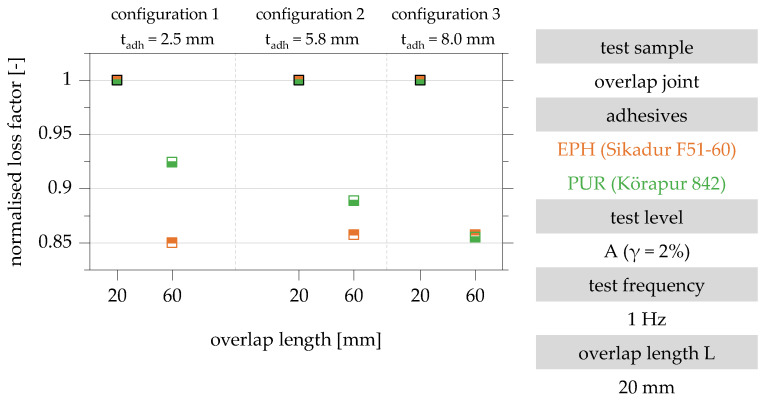
Evaluation of the influence of the overlap length on the loss factor of the overlap joint configurations 1, 2 and 3 with overlap lengths 20 and 60 mm depending on the adhesive used; representation normalised to the loss factor of the test specimens with overlap length 20 mm for the adhesives EPH and PUR based on [22].

In addition to the general difference in the damping behaviour of the two adhesives, the influence of the overlap length on the damping properties of the overlap joints made with the EPH and PUR adhesives is quantified in the following. Figure 13 shows the loss factors of the overlap joint configurations 1, 2 and 3 for the adhesives EPH and PUR normalised to the overlap length of 20 mm.

With increasing adhesive layer thickness, an approximately linear increase in the influence of the overlap length on the loss factor can be observed for the PUR adhesive. As a result of increasing the adhesive layer thickness to 8.0 mm (configuration 3), the loss factor normalised to an overlap length of 20 mm decreases by approx. 14% for both adhesives EPH and PUR [22].

### 4.5. Analytical Description of the Damping Properties of Adhesively Bonded Overlap Joints

#### 4.5.1. Definition of Dimensionally Dependent Influencing Factors

The analysis of the results of the experimental investigation in the previous sections has shown that the damping properties of adhesively bonded overlap joints depend on the general specimen geometries, the resulting geometric dimensions of the adhesive layer and the specific conditions of the loading during the test. The corresponding properties of the mentioned influencing factors are summarised in Table 3. According to [33], the loss factor is also included in the list of influencing factors as a target variable to be described.

By applying the Pi Theorem (see Section 2.3), the total of seven influencing factors and the two basic dimensions result in 7−2 = 5 dimensionless parameters, which are determined as given in Table 4.

The loss factor is dimensionless and can, therefore, be used as an independent, dimensionless parameter П_1_. П_2_ is derived from the adhesive layer thickness t_adh_ and the overlap length L and thus forms a ratio that is common in adhesive bonding technology to characterise the decisive dimensions of the adhesive layer. The adhesive layer width B, which has not been considered so far, is set in relation to the maximum shear deformation in parameter П_3_. The geometries of the adhesive layer are thus considered by the parameters П_2_ and П_3_. For the derivation of parameter П_4_, a parameter that is also known in adhesive bonding technology is used. The quotient of the maximum shear deformation and the adhesive layer thickness has already been introduced as a dimensionless shear strain γ and can, therefore, be used in the context of dimensional analysis. Finally, the parameter П_5_ considers the test frequency f and the respective test period in the form of time t [22].

#### 4.5.2. Identification of the Functional Relationship of the Dimensionless Parameters

**Figure 14:**
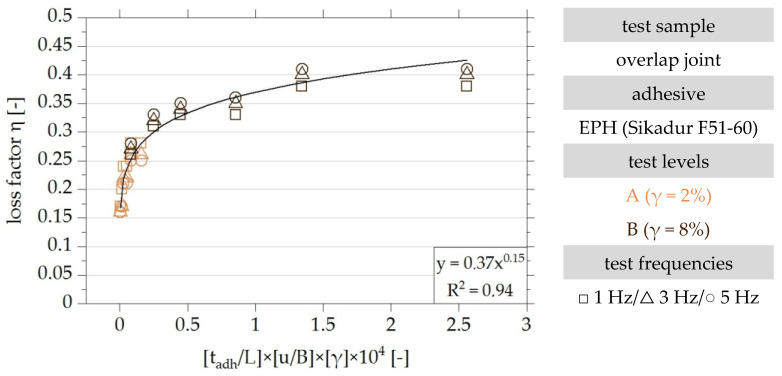
Dimensionless visualisation of the results of the experimental investigation of overlap joints made with the EPH adhesive and their approximation by a logarithmic regression function, taking into account the dimensionless parameters Π_2_, Π_3_ and Π_4_ based on [22].

The functional relationship of the identified dimensionless parameters is identified iteratively. In the first step, the results of the experimental tests on overlap joints made with the adhesive EPH are plotted in Figure 14 over the product of the dimensionless parameters П_2_, П_3_ and П_4_. Thus, it is assumed that the loss factor is linearly proportional to the parameters П_2_, П_3_ and П_4_. These are multiplied by a factor of 10^4^ for better visualisation.

The influence of the level of shear strain in the range between 2% and 8% on the loss factor cannot be determined with certainty on the basis of the test results. However, it can be seen that the loss factor increases with an increasing shear strain of the adhesive layer. For this reason, a linear influence of the shear strain on the loss factor is assumed as a first approximation.

To evaluate the quality of the identified functional relationship, Figure 14 also shows a regression curve as an exponential function. The parameter of determination of the exponential function given is R^2^ = 0.94 and thus confirms the assumption that the functional relationship of the test results can be described in a good approximation by a linear proportional relationship of the parameters П_2_, П_3_ and П_4_. Investigations documented [22] show that the approximation of the functional relationship cannot be improved by varying the exponent of parameter П_4_ between 0.75 and 2.

**Figure 15:**
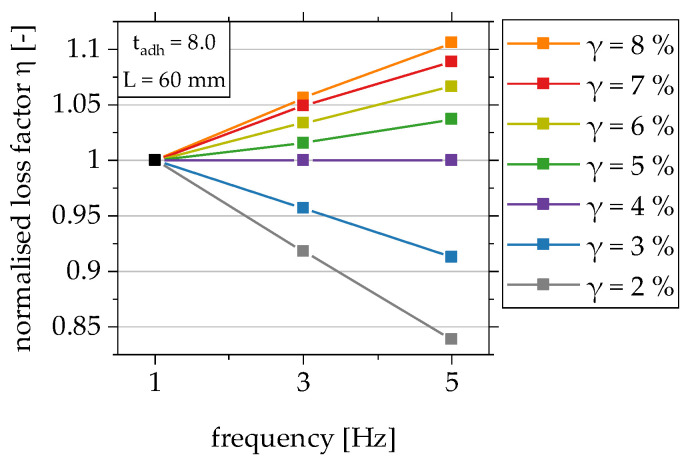
Analysis of the functional relationship between the shear strain level and the influence of the test frequency on the loss factor in the range of shear strain of the adhesive layer from 2% to 8% for overlap joint configuration 3 with overlap length 60 mm; plot normalised to the frequency 1 Hz [22].

Lastly, the influence of the test frequency is considered in the functional relationship by the dimensionless parameter Π_5_. The influence of the test frequency on the loss factor, which depends on the shear strain of the adhesive layer, was investigated in [22]. The correlation between the shear strain level and the influence of the test frequency on the loss factor is so far only known for the shear strain levels 2% and 8%. The results on the influence of the test frequency as a function of the shear strain level are, therefore, extended by exemplary investigations on overlap joint configuration 3 with the overlap lengths 20 mm and 60 mm. The experimental set-up and procedures are described in Section 2.2.3. The shear strain level is increased in 1% increments. The results normalised to the frequency of 1 Hz are shown in Figure 15 as an example of the overlap length of 60 mm.

**Figure 16:**
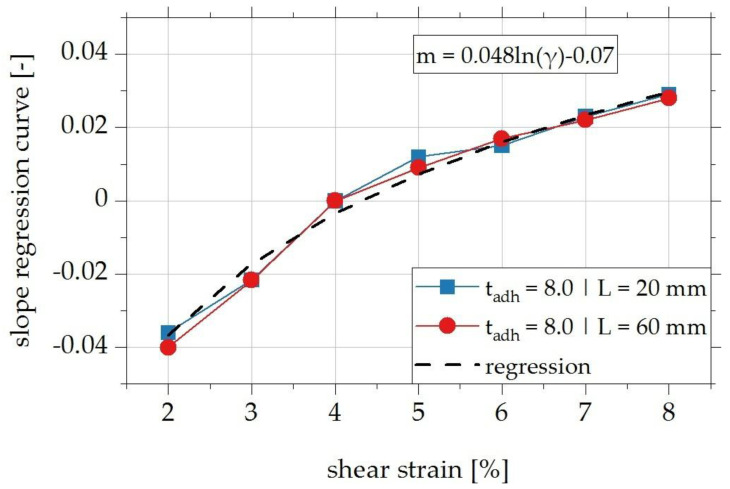
Slope of the regression curve to describe the influence of the test frequency on the damping properties of overlap joints adhesively bonded with the adhesive EPH as a function of the shear strain level; approximation of the experimental data by a logarithmic regression function [22].

It can be seen that the loss factor for all investigated shear strain levels changes linearly with an increase in frequency. Based on these results, the approach is to consider the functional dependence of the loss factor on the test frequency via the slope of the regression line in the dimensional analysis. For this purpose, the slopes of the linear regression lines of the test results shown in Figure 15 are first determined separately for each shear strain level and plotted separately in Figure 16 for the two investigated overlap lengths above the respective value of the shear strain. The mean value curve can be approximated by the logarithmic regression curve shown in Figure 16 with the given functional equation in Equation (5) [22].

The change of the loss factor Δη due to an increase in the test frequency can be taken into account based on Figure 16 via Equation (5) in the dimensionless, functional relationship. In this case, the reference frequency f_ref_ is set to 1 Hz.

(5) $$m=\frac{\Delta \eta }{\left(f-f_{\mathrm{ref}}\right)t}=\frac{\Delta \eta }{\Delta f\times t}\to \Delta \eta =m\times \Delta f\times t$$

The functional relationship of the dimensionless parameters Π_2_ to Π_5_ can be written as follows (see Equation (6)):

(6) $$F\;\left(\eta ,\;\frac{t_{\mathrm{adh}}\times u}{L\times B}\times \gamma \times \left(1+m\times \Delta f\times t\right)\right)=0$$

**Figure 17:**
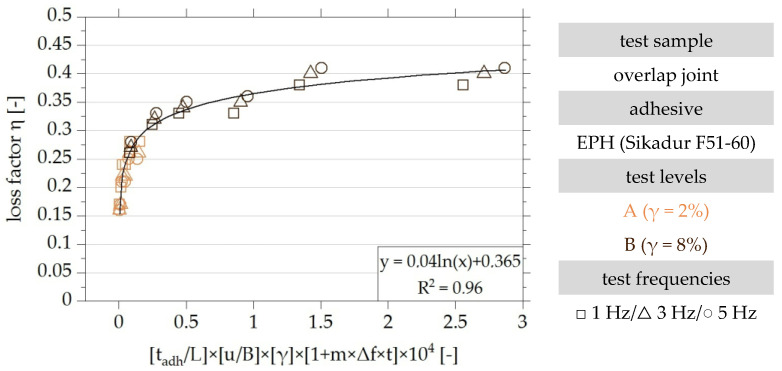
Dimensionless visualisation of the results of the experimental investigation of overlap joints made with the EPH adhesive and their approximation by a logarithmic regression function, taking into account the dimensionless parameters Π_2_, Π_3_, Π_4_ and Π_5_ based on [22].

In order to evaluate the quality of the curve fitting Figure 17 plots the experimental results over the functional relationship documented in Equation (6).

The functional relationship of all test results can be optimised by considering all identified influencing factors and can be approximated with very good accuracy by the logarithmic regression function documented in Figure 17 with a parameter of determination of R^2^ = 0.96 [22].

## 5. Conclusions

The investigations on the influence of geometry and test conditions on the damping properties (loss factors) of adhesively bonded overlap joints bonded can be summarised as follows:

The influence of the test frequency on the loss factor depends on the stress level of the adhesive layer. In the approximately linear viscoelastic range of the adhesive (γ ≤ 2%), an increase in frequency leads to a decrease in the dissipative properties of the bonded samples. As a result of an increase in the level of strain (γ ≥ 8%), an inverse relationship can be observed, and the loss factor increases with an increase in the test frequency. In general, the influence of the test frequency on the loss factor of the overlap joint is of minor relevance.

An increase in the overlap length from 20 mm to 60 mm leads to a decrease of the loss factor by 14% in the mean values of all considered adhesive layer thicknesses. An increase in the adhesive layer thickness from 2.5 mm to 5.8 mm results in an increase in the loss factor of up to 41%. A further increase in the loss factor due to an increase in the adhesive layer thickness cannot be observed. The influence of the adhesive layer thickness on the loss factor also increases with increasing adhesive layer stress.

In summary, an analysis of the influence of the geometry parameters of the investigated test specimens shows that the damping properties of adhesively bonded overlap joints can be enhanced by increasing the adhesive layer thickness and reducing the overlap length. It follows that the structural design of adhesively bonded joints of dynamically stressed structures must be considered against the background of the geometric dimensions of the adhesive layer [20].

Table 5 provides an overview of the main results, taking the interactional relationships of the investigated parameters into account. The indicated arrows show the change of the considered parameter (↑: increase) as well as the effects on the loss factor (↓: reduction, ↑: increase).

The functional relationships of all the test results shown can be determined by dimensional analysis. An analytical determination of the loss factor, taking into account all identified influencing factors, is enabled by derived regression functions with a high coefficient of determination (R^2^ = 0.96).

The developed methodology offers the advantage that the damping properties of bonded test specimens can be determined on the basis of a small number of tests, even for non-investigated test and geometry boundary conditions. The development of numerical material models with which the damping properties of the adhesives can be mapped is often very complex. Experimental investigations are also required to validate the models. For a practical design of structures, these procedures are too complex and often cannot be implemented by the designing engineer. A graphical prediction for estimating the damping properties of bonded joints represents an efficient engineering approach.

Therefore, the developed method forms the basis for the consideration of the damping properties of adhesively bonded joints, taking into account defined validity limits in the context of the practical design of structures.

## Figures and Tables

**Table 1:** Test parameters and test series of the experimental investigations of the damping behaviour of the adhesively bonded overlap joints based on [22].

Configuration	Adhesive	Adhesive Layer Thickness t_adh_ [mm]	Overlap Length L [mm]	Test Level	Frequency [Hz]
1	EPH/ PUR	2.5	20	A (γ ± 2%)/B (γ ± 8%)	1/3/5
			60		
2		5.8	20		
			60		
3		8.0	20		
			60		

**Table 2:** Summary of the loss factors determined for the overlap joint configurations 1, 2 and 3 depending on the overlap length, the test level and the frequency.

Configuration	Adhesive, t_adh_ [mm]	Overlap Length L [mm]	Test Level	Loss Factor at Frequency					
				1 Hz	*CV*	3 Hz	*CV*	5 Hz	*CV*
1	EPH, 2.5	20	A (γ ± 2%)	0.20	*0.04*	0.17	*0.10*	0.17	*0.13*
		60		0.17	*0.03*	0.16	*0.09*	0.16	*0.09*
2	EPH, 5.8	20		0.28	*0.02*	0.26	*0.04*	0.25	*0.09*
		60		0.24	*0.03*	0.22	*0.06*	0.21	*0.05*
3	EPH, 8.0	20		0.28	*0.02*	0.26	*0.05*	0.25	*0.05*
		60		0.24	*0.08*	0.22	*0.10*	0.21	*0.06*
1	EPH, 2.5	20	B (γ ± 8%)	0.31	*0.03*	0.32	*0.02*	0.33	*0.03*
		60		0.26	*0.02*	0.27	*0.04*	0.28	*0.06*
2	EPH, 5.8	20		0.38	*0.02*	0.40	*0.02*	0.41	*0.04*
		60		0.33	*0.03*	0.34	*0.03*	0.35	*0.03*
3	EPH, 8.0	20		0.38	*0.02*	0.40	*0.03*	0.41	*0.03*
		60		0.33	*0.02*	0.35	*0.05*	0.36	*0.02*

**Table 3:** Influencing factors for characterising the damping properties of bonded overlap joints based on [22].

Influencing Factors	Symbol	Dimension
loss factor	η	[1]
adhesive layer thickness	t_adh_	[L]
overlap length	L	[L]
adhesive layer width	B	[L]
maximum shear deformation of the adhesive layer	u	[L]
frequency	f	[T^−1^]
time	t	[T]

**Table 4:** Defined dimensionless parameters Π_1_ to Π_5_ based on [22].

Dimensionless Parameter	Functional Form
Π_1_	η
Π_2_	t_adh_/L
Π_3_	u/B
Π_4_	u/t_adh_ = γ
Π_5_	f × t

**Table 5:** Influence and interactional relationships of the investigated parameters on the loss factor of adhesively bonded overlap joints.

Parameter			Interactional Relationships		Influence on η
frequency	f	↑	γ	low strain level | γ ≤ 2%	↘
				increasing strain level | γ ≥ 8%	↗
overlap length	L	↑	-	-	↓
adhesive layer thickness	t_adh_	↑	γ	increasing influence for higher strain level	↑

## Data Availability

The data presented in this study are available upon request from the corresponding author.
